# Multifactor Regulation of the MdtJI Polyamine Transporter in *Shigella*

**DOI:** 10.1371/journal.pone.0136744

**Published:** 2015-08-27

**Authors:** Adriano Leuzzi, Maria Letizia Di Martino, Rosaria Campilongo, Maurizio Falconi, Marialuisa Barbagallo, Lucia Marcocci, Paola Pietrangeli, Mariassunta Casalino, Milena Grossi, Gioacchino Micheli, Bianca Colonna, Gianni Prosseda

**Affiliations:** 1 Istituto Pasteur-Fondazione Cenci Bolognetti, Dipartimento di Biologia e Biotecnologie “C. Darwin”, Sapienza Università di Roma, Via dei Sardi 70, 00185, Roma, Italy; 2 Laboratorio di Genetica Molecolare e dei Microrganismi, Scuola di Bioscienze e Medicina Veterinaria, Università di Camerino, Via Gentile III da Varano, Camerino, Italy; 3 Dipartimento di Biochimica, Sapienza Università di Roma, P.le A. Moro 5, 00185, Roma, Italy; 4 Dipartimento di Scienze, Università Roma Tre, Viale G. Marconi 446, 00146, Roma, Italy; 5 Istituto di Biologia e Patologia molecolari CNR, P.le A. Moro 5, 00185, Roma, Italy; Centre National de la Recherche Scientifique, Aix-Marseille Université, FRANCE

## Abstract

The polyamine profile of *Shigella*, the etiological agent of bacillary dysentery in humans, differs markedly from that of *E*. *coli*, its innocuous commensal ancestor. Pathoadaptive mutations such as the loss of cadaverine and the increase of spermidine favour the full expression of the virulent phenotype of *Shigella*. Spermidine levels affect the expression of the MdtJI complex, a recently identified efflux pump belonging to the small multi-drug resistance family of transporters. In the present study, we have addressed the regulation of the *mdtJI* operon in *Shigella* by asking which factors influence its expression as compared to *E*. *coli*. In particular, after identifying the *mdtJI* promoter by primer extension analysis, *in vivo* transcription assays and gel-retardation experiments were carried out to get insight on the silencing of *mdtJI* in *E*. *coli*. The results indicate that H-NS, a major nucleoid protein, plays a key role in repressing the *mdtJI* operon by direct binding to the regulatory region. In the *Shigella* background *mdtJI* expression is increased by the high levels of spermidine typically found in this microorganism and by VirF, the plasmid-encoded regulator of the *Shigella* virulence regulatory cascade. We also show that the expression of *mdtJI* is stimulated by bile components. Functional analyses reveal that MdtJI is able to promote the excretion of putrescine, the spermidine precursor. This leads us to consider the MdtJI complex as a possible safety valve allowing *Shigella* to maintain spermidine to a level optimally suited to survival within infected macrophages and, at the same time, prevent toxicity due to spermidine over-accumulation.

## Introduction

Polyamines, such as putrescine, spermidine, and cadaverine, are aliphatic polycationic molecules found in all cells. They are necessary for normal cell growth and have been associated with a wide variety of physiological processes, primarily those involving nucleic acids, such as DNA synthesis, transcription and translation [1]. Among polyamines, a peculiar role is played by putrescine, the precursor of most polyamines [2]. In *E*. *coli* putrescine is the predominant polyamine, followed by spermidine and cadaverine, while spermine is absent. The intracellular polyamine content depends on both *de novo* synthesis from decarboxylation of precursor aminoacids and uptake from the outer environment mediated by specific transporters [2].

In *E*. *coli* polyamine transport relies on several systems [3]. Two major uptake systems, functioning at neutral pH, belong to the family of the ABC transporters. In particular, a spermidine-preferential uptake system consists of the PotA, PotB, PotC and PotD proteins and is encoded by the *potA-D* operon, while a putrescine-specific uptake system is constituted by the PotF, PotG, PotH and PotI proteins and is encoded by the *potF-I* operon. Two additional polyamine transporters, PotE and CadB, each constituted by a single protein endowed with twelve transmembrane domains, act as antiporters of putrescine/ornithine and, respectively, cadaverine/lysine. The genes for PotE and CadB are located within the *cadBA* and *speF-potE* operons, which encode also ornithine decarboxylase and lysine decarboxylase, and are induced by acid pH. Besides excreting putrescine and cadaverine, PotE and CadB also act as uptake proteins for the same polyamines at neutral pH. Two additional putrescine importers have been recently identified, PuuP, which is one of the proteins involved in the putrescine utilization pathway [4], and YeeF which is required for surface motility induced by extracellular putrescine [5].

It has become increasingly evident that, in addition to core physiological functions, polyamines are crucial also to the virulence phenotype of many bacterial pathogens [6]. These microorganisms have developed their own strategies to exploit polyamines to optimize their survival within the host. *Shigella*, the etiological agent of bacillary dysentery in humans [7], is an interesting example since during the evolutionary transition from its innocuous ancestor, *E*. *coli*, its polyamine profile has undergone drastic changes. *Shigella* was traditionally divided into four species: *S*. *flexneri*, the prevalent one causing large endemic infections; *S*. *dysenteriae*, responsible for deadly outbreaks; *S*. *sonnei*, associated with mild infections in industrial countries; and *S*. *boydii*, endemic in the Indian sub-continent [8]. However, comparative genomics studies have demonstrated that, rather than forming a distinct genus, *Shigella* strains belong to the extremely diverse *E*. *coli* species and have derived repeatedly from multiple *E*. *coli* strains by convergent evolution involving both gain and loss of genes [9]. *Shigella* has a highly specialized invasive system that enables the bacteria to penetrate into epithelial cells and macrophages, survive within them and eventually lead to the inflammatory destruction of the colonic mucosa. The underlying pathogenic process is a complex one, requiring the coordinated expression of several genes located both on the chromosome and on a large virulence plasmid (pINV). Outside the human host, transcription of pINV genes is strongly repressed by the nucleoid protein H-NS [10]. Within the host, activation of the invasion genes occurs through a regulatory cascade involving the plasmid encoded regulators VirF, VirB and MxiE [11]. The primary event after the shift to the human host temperature is the synthesis of VirF, an AraC like activator whose promoter is silenced by H-NS at low temperature as a direct consequence of its increased intrinsic curvature [12]. The full expression of the plasmid invasive genes is achieved by a delicate interplay among specific regulators, nucleoid proteins, and sRNA molecules [13,14,15].

In *Shigella* polyamines have an antagonistic effect on the invasive process. While spermidine accumulation has been shown to correlate with increased survival during infection of macrophages [16,17], the lack of cadaverine production is known to increase the pathogenic potential of the bacterium in host tissues [18]. The increased level of spermidine depends on the absence of spermidine acetyltransferase (SAT), the enzyme, which converts spermidine into its inert form, contrasting the toxicity of high spermidine concentrations in *E*. *coli*. The lack of cadaverine is determined by the inactivation of the genes involved in the biosynthesis and transport of cadaverine. In both cases gene silencing is the result of convergent evolution processes [18,19].

Recently it has been shown that in *E*. *coli* spermidine can be excreted also by the MdtJI efflux pump [20] but only when the polyamine over-accumulates within the cell and in the extracellular environment for more than 24 hours. The MdtJI complex belongs to the small multidrug resistance (SMR) family of drug exporters [21]. It is encoded by the *mdtJI* operon which, under physiological conditions, is expressed at a very low level. The two protein components of the pump, MdtJ and MdtI (121 and 109 aminoacids, respectively), have four transmembrane segments, and most of the functional aminoacid residues face the cytoplasm [20], an organization recalling that of other polyamine excretion proteins, such as PotE and CadB [22]. In the present work, we asked whether an *mdtJI* operon is expressed in *Shigella* and investigated on its role in polyamine exchange and on the factors controlling its expression. We find that repression of the *mdtJI* operon is mediated by H-NS both in *Shigella* an in *E*. *coli*. However, in *Shigella* we find that the expression of the *mdtJI* operon is increased by high levels of spermidine and by the presence of the primary regulator VirF. Moreover, we find that bile promotes *mdtJI* expression and that, in a polyamine-free medium, MdtJI confers *Shigella* the ability to stimulate the excretion of putrescine, the precursor of spermidine.

## Materials and Methods

### Bacterial strains and growth conditions

Bacterial strains are listed in Table 1. M90T is a *S*. *flexneri* serotype 5 strain whose genome has been completely sequenced (GenBank CM001474.1). Strain M90T Ed carries a deletion of the *speE* gene coding for spermidine synthase [16]. Strains M90T JId, carrying a deletion of the *mdtJI* operon, and M90T Fd, carrying a deletion of the *virF* gene, have been constructed using the one-step method of gene inactivation [23] by transforming M90T pKD46 with amplicons obtained using plasmid pKD13 as template and the oligo pairs *JIdF/JIdR* (*mdtJI* deletion) or *dff/dfr* (*virF* deletion) (S1 Table). Under the experimental conditions used no difference in growth rate was observed between M90T and its *mdtJI* and *virF* derivatives. M90T Hd and ULS504 were obtained by transducing the *hns118* allele of TP504 into M90T and MG1655, respectively. P1 transduction was carried out as previously described [13]. Strain M90T virF-FT was obtained by introducing the 3xFLAG tag sequence at the C-terminus of the pINV-encoded *virF* gene. A PCR product obtained using oligos *ftFF*/*ftFR* (S1 Table) and plasmid pSUB11 (Table 1) as template was introduced into M90T pKD46, and recombinants selected according to Uzzau et al. [24].

**Table 1:** Bacterial strains and plasmids used in this study.

	Relevant characteristics	Source and/or reference
**Strain**		
M90T	Wild-type *S*. *flexneri* serotype 5; LDC^-^ ^a^	[28]
MG1655	*E*. *coli* K12, *F* ^*-*^, ʎ ^*-*^, *ilvG* ^*-*^, *rfb-50*, *rph-1*	ATCC47076^a^
ULS153	Same as MG1655 but Δ*lacZYA;* Km^r^	[16]
TP504	*F* ^*-*^ *leuB6 serB1203 thi-1 tonA21 lacY1 supE44 zch506*::*Tn10 zdd230*::*Tn9 Δ(bgl Y-irk-drs-drc)*	[27]
M90T JId	Same as M90T but Δ *mdtJI* Km^r^	This study
M90T Fd	Same as M90T but Δ *virF*; Km^r^	This study
M90T Ed	Same as M90T but Δ *speE*; Km^r^	[16]
M90T Hd	Same as M90T but *hns*_118_; Tc^r^	This study
M90T *virF*-FT	Same as M90T but *virF 3xFLAG tag*	This study
ULS504	Same as MG1655 but *hns*_118_ Tc^r^	This study
**Plasmid**		
pGEM-T Easy	TA cloning vector	[29]
pGEM-3Z	Equivalent replicative form to pGEM-T Easy	GenBank nr X65304
pULS85	pGEM-T-easy derivative containing the *mdtJI* promoter region, Ap^r^	This study
pULS88	pGEM T-Easy derivative containing a functional *mdtJI* operon,; Ap^r^	This study
pMYSH6504	pBR322 derivative containing the *Shigella virF* gene, Ap^r^ Km^r^	[26]
pDIA510	pBR322 derivative containing the *E*. *coli hns* gene; Ap^r^	[27]
pULS37	pACY184 derivative containing the *speG* gene with its promoter region; Tc^r^	[16]
pULS13	pACY184 derivative containing the *speG* gene under P_tac_ control: Tc^r^	[16]
pKD13	Template plasmid carrying a Km^r^ gene with FLP recognition target sequence	[23]
pKD46	Red recombinase expression plasmid	[23]
pSUB11	Template plasmid carrying a 3xFLAG sequence and FRT sites flanking a kanamycin resistance cassette	[24]
pRS415	*lacZYA* transcriptional fusion vector	[25]
pJI*lac*-3	pRS415 derivative carrying 593 bp of the *mdtJI* region	This study
pJI*lac*-2	pRS415 derivative carrying 510 bp of the *mdtJI* region	This study
pJI*lac*-1	pRS415 derivative carrying 370 bp of the *mdtJI* region	This study

All strains are indicated by their original laboratory name

^(a)^ ATCC, American Type Culture Collection.

Bacterial cells were routinely grown at 37°C in Luria-Bertani (LB) broth. When required cells were grown in polyamine-free M9 complete medium (M9 minimal medium supplemented with 10 μg/ml thiamine, 0.2% glucose, 0.5% casamino acids and 10 μg/ml nicotinic acid). Solid media contained 1.6% agar. Antibiotics were included at the following final concentrations: ampicillin (Ap) 100 μg/ml; chloramphenicol (Cm) 30 μg/ml; kanamycin (Km) 30 μg/ml; tetracyclin (Tc) 5 μg/ml. Sensitivity assays to sodium deoxycholate and bile salts have been performed by spot tests on LB agar plates (cultures grown to OD_600_ 0.6) containing 2.5 and 5 mg/ml deoxycholate or 6 and 9 mg/ml bile salts. The ability of MdtJI to confer antibiotic resistance was assayed by agar diffusion testing (E strip on Mueller Hinton Agar plates) in order to determine the Minimum Inhibitory Concentration (MIC) for nalidixic acid (NA), fosfomycin (F), chloramphenicol (C), tetracycline (Tc), gentamicin (CN), erythromycin (E), rifampicin (RD), spectinomycin (SPC), streptomycin (S), trimethoprim (Tm), ampicillin (Ap), kanamycin (K), sulfamethoxazole (SMX), and ciprofloxacin (CIP).

### Plasmid construction

Plasmid pULS85 and pULS88, containing the *mdtJI* regulatory region or the entire *mdtJI* operon, were constructed by cloning, into pGEM-T Easy, DNA fragments obtained by PCR with the oligo pair *mdF*/*mdR* or *JIF*/*JIR* (S1 Table) and total DNA of *S*. *flexneri* M90T as template.

Plasmids pJI*lac*-3, pJI*lac*-2 and pJI*lac*-1 (Table 1), carrying fusions with the *lacZ* reporter gene, were constructed by cloning different PCR-generated fragments of the *mdtJI* regulatory region into the multi-cloning site of the *lacZYA* transcriptional fusion vector pRS415 [25]. In particular, (a) plasmid pJI*lac*-3, containing all the predicted H-NS boxes, was generated by cloning a 593 bp fragment obtained using the oligo pair *JIfusF*/*JIfusR3*; (b) plasmid pJI*lac*-2, containing the two promoter proximal predicted H-NS boxes, was generated by cloning a 510 bp fragment obtained using the oligo pair *JIfusF*/*JIfusR2*; and (c) plasmid pJI*lac*-1, containing the single H-NS box at the promoter, was generated by cloning a 370 bp fragment obtained using the oligo pair *JIfusF*/*JIfusR1*. Sequences of the oligo pairs are reported in S1 Table. PCR reactions were performed with high-fidelity Taq polymerases and M90T DNA as template. The cloned fragments were verified by sequencing.

Plasmids pMYSH6504 and pDIA510 are both pBR322 derivatives containing, respectively, the *virF* gene of the *S*. *flexneri 2a* pINV [26] and the *hns* gene of *E*. *coli* [27]. pULS37 and pULS13 are pACYC184 derivatives containing the *E*. *coli speG* gene under the control of its regulatory region or of the P_tac_ promoter, respectively [16].

### General procedures

Plasmid DNA extraction, DNA transformation, cloning, restriction, electrophoresis, purification of DNA fragments and sequencing were carried out as described previously [29,30]. PCR reactions were performed using Dreamtaq DNA polymerase or Pfu Taq DNA polymerase when a higher fidelity was required. All oligonucleotides used in this study are listed in S1 Table and have been designed mainly on the basis of the genomic sequence of *S*. *flexneri* M90T (GenBank CM001474.1) or *E*. *coli* K12 MG1655 (GenBank NC_000913.3).

β-galactosidase assays were performed as previously described [31] on sodium dodecyl sulfate-chloroform-permeabilized cells grown in M9 complete medium (to OD_600_ 0.5–0.6) supplemented with ampicillin.

### Immunoblotting

For the immunodetection of VirF, we used strain *S*. *flexneri* M90T *virF*-FT carrying a *virF* 3xFLAG tag fusion (Table 1) or its derivative complemented with a s*peG* recombinant plasmid (pULS13). Equal amount of proteins was extracted from strains grown at OD_600_ 0.2 or 0.6, separated on 15% SDS-PAGE gels and transferred onto nylon C-extra membranes. Membranes were blocked for 1 h with 5% dry skimmed milk in PBS-T (PBS with 0.1%, Tween20). Incubation with primary anti FLAG mouse antibodies (1:750) was performed overnight at 4°C in PBS-T containing 2% dried skimmed milk. Membranes were washed and incubated at room temperature for 1 h with a secondary anti-mouse horseradish-peroxidase-conjugated antibody (1:5,000) in PBS-T. After washing with PBS-T, membranes were developed for 5 min in ECL and visualized on a ChemiDoc XRS+ system. The relative amount of protein was quantified using the Image lab software (3.0).

### Polyamine quantification

Bacteria were grown in M9 complete medium to 10^8^ cells/ml and pelleted by centrifugation. An aliquot of the supernantants was saved for polyamine extraction and bacterial cell pellets were first resuspended in PBS and then disrupted by sonication. Polyamines were extracted from supernatants and cell lysates with 3% percloric acid containing 5mM 1,6-diaminehexane as polyamine internal standard. After derivatization with dansylchloride the simultaneous fluorimetric determination of intracellular polyamines was performed by reverse-phase high-performance liquid chromatography and polyamines were quantified as described previously [32]. The polyamine concentration was normalized to cell number and expressed as nmol/10^8^ cells (cell lysate samples) or nmol/ml supernant.

### Northern analysis

Bacterial strains were grown at 37°C to OD_600_ 0.4 in LB broth supplemented with the appropriate antibiotic. Equivalent aliquots of total RNA, extracted as previously described [33], were denatured at 100°C for 5 min in the presence of 2M formaldehyde and 50% formammide, and then separated on agarose gel. The relative amounts of RNA loaded in each lane were estimated by visualization of the rRNA by ethidium bromide staining. Gels were then electroblotted onto N+ membranes and hybridized as previously described [12] using a *α-* ^32^P-labelled *mdtJI* specific probe (a 391 bp fragment obtained by amplifying total DNA of MG1655 with the oligo pair *mtpF*/*mtpR*).

### Real Time PCR

Total RNA purification and cDNA synthesis were performed as previously described [34]. Real time PCR was performed using a 30 μl reaction mix containing 2 μl cDNA. At least three wells were run for each sample. The amount of *mdtJI* transcripts was analysed using the 2^-ΔΔCt^ method [35] and the results were indicated as n-fold increase relative to the reference sample. The ΔCt-values have been considered in the Student’s t test to determine whether datasets of relative gene expression were significantly different from that detected in a chosen calibrator. Primers for the *nusA* transcript, used as endogenous control, and for the *mdtJI* and *virF* transcripts were designed with the aid of the Primer Express software v2. 0 and experimentally validated for suitability for the 2^-ΔΔCt^ method. The following oligos (S1 Table) were used: *mJIf* /*mJIr* for *mdtJI*, *nusAF*/*nusAR* for *nusA* and *virFQF*/*virFQR* for *virF* transcript.

### Primer extension

Total RNA from *E*. *coli* pULS85 grown to OD_600_ 0.6 was extracted by a modified hot-phenol method and quantified spectrophotometrically as described [33]. The primers *mPE1* and *mPE2* were 5’-end labelled with [γ-^32^P]dATP using T4 polynucleotide kinase and hybridized with 50 μg total RNA as previously described [36]. Reverse transcription experiments were carried out at 42°C and the resulting cDNAs were run on denaturing 6% polyacrylamide gels, along with a sequencing ladder that was generated using the same primer and pULS85 as templates. Sequencing reactions were performed with a T7 polymerase-based DNA sequencing procedure and [γ-^32^P]dATP.

### Electrophoretic Mobility Shift Assay (EMSA)

The 588 bp fragment containing the *mdtJI* promoter (-229 to +359) was obtained by PCR amplification of pULS85 DNA using the primers *mdF*/*mdR*. The amplicon was end-labelled with ^32^P-dATP by a fill-in reaction with the Klenow DNA polymerase fragment as previously described [37]. The reaction mixture contained, approximately 5 ng of DNA fragment, 50 ng poly(dI-dC) as competitor DNA and the indicated concentrations of purified H-NS in a total volume of 15 ul. EMSA was carried out essentially as previously described [38]. Samples were subjected to electrophoresis on 7% polyacrylamide gels in TAE buffer (Tris-HCI 40 mM pH 7.4, sodium acetate 5 mM, EDTA 1 mM). H-NS was prepared according to the protocol previously described [36].

### In silico analyses

Genome comparison was carried out using the NCBI BLAST online tools (http://blast.ncbi.nlm.nih.gov/Blast.cgi). We used Digital Science 1D software for densitometric quantitations. The -10 and -35 boxes in the *mdtJI* promoter were predicted according to the “Neural Network Promoter Prediction” on-line tool (http://www.fruitfly.org/seq_tools/promoter.html). The identification of putative H-NS binding boxes was performed using the “Promoter Analysis” tool provided by the “Virtual footprint” on line service (http://www.prodoric.de/vfp/) [39].

Computer-generated predictions of intrinsic DNA curvature were obtained with D.I.C.E. (DNA Intrinsic Curvature Evaluator), a software developed by one of the authors (GM) for the analysis of sequence-mediated DNA curvature [12,33]. In essence, the software is an implementation of the CURVATURE algorithm [40] on Windows platforms which generates various quantitative curvature estimates.

## Results

### Transcriptional regulation of the *mdtJI* operon in *Shigella*

MdtJI is an efflux pump belonging to the small multi-drug resistance (SMR) transporter family [21]. In *E*. *coli* it is expressed at very low level under physiological conditions, but it has been shown to promote spermidine excretion when cloned in a multicopy expression vector and in the presence of high spermidine concentrations [20]. Banking on this observation, we investigated whether the MdtJI complex is present in *Shigella*, a bacterial pathogen which has a high intracellular level of spermidine [16] and shares common evolutionary roots and high genome homology with *E*. *coli* [9] but undergoes extensive gene decay as compared to its ancestor [41].

**Fig 1:**
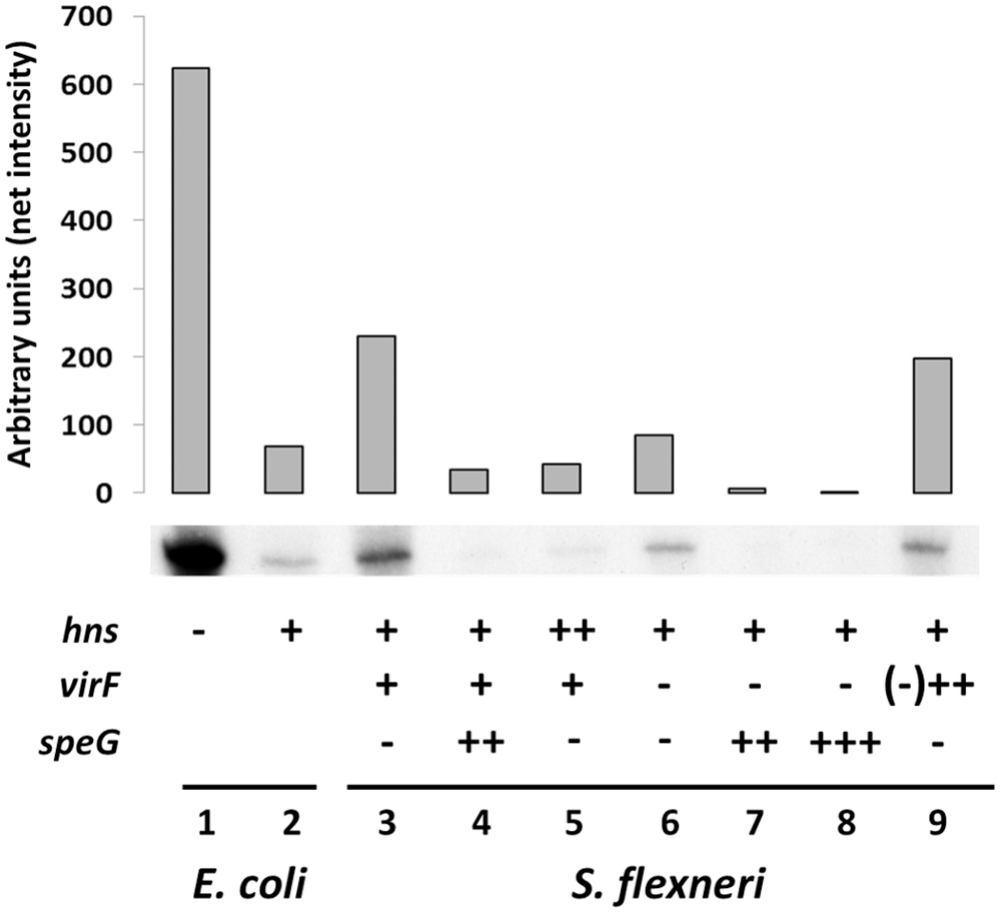
H-NS, VirF and spermidine regulate *mdtJI* expression. Northern analysis of *mdtJI* expression performed with a α-^32^P-labelled *mdtJI* probe and total RNA extracted from the following strains: *E*. *coli* K12 ULS504 (MG1655Δ*hns*, lane 1), MG1655 (lane 2), *S*. *flexneri* M90T wt (*hns* ^+^ *virF* ^+^ *speG* ^-^; lane 3), M90T pUL37 (lane 4), M90T pDIA510 (lane 5) M90T Fd (Δ*virF*; lane 6), M90T Fd pULS37(lane 7), M90T Fd pULS13 (lane 8), M90T Fd pMYSH6504 (lane 9). All strains were grown at 37°C in LB medium. The presence of a recombinant plasmid is indicated by ++, except in the case of pULS13 (P_tac_ *speG*) where it is indicated by +++. Upon autoradiography the hybridization signals were quantified by densitometric analysis.

By comparing the genome of *Shigella* with that of *E*. *coli* we find that the genes coding for the *mdtJ* and *mdtI* subunits are conserved in *Shigella* spp. (*S*. *flexneri*, *S*. *boydi*, *S*. *dysenteriae* and *S*. *sonnei*) and map at the same location, i.e between the *ydgD* and *tqsA* genes. We have recently reported that, as compared to *E*. *coli*, *Shigella* accumulates spermidine due to a pathoadaptive mutation in the gene (*speG*) encoding acetylspermidine synthase, and that the introduction of a functional *speG* allele in *S*. *flexneri* induces a 3-fold reduction of intracellular spermidine [16]. We asked whether the increased spermidine content of *Shigella* influences the expression of the *mdtJI* operon. To this end, we compared *mdtJI* transcription in *Shigella* strains expressing or lacking *speG*. Equivalent amounts of total RNA extracted from *S*. *flexneri* M90T (wt) and from its derivative complemented with a functional *speG* gene (M90T pULS37) were denatured, separated on agarose gels and hybridized with a *mdtJI*-specific probe. Northern analysis reveals that in the *speG*-complemented strain *mdtJI* expression is strongly reduced as compared to the wt (Fig 1, lanes 3 and 4).

**Fig 2:**
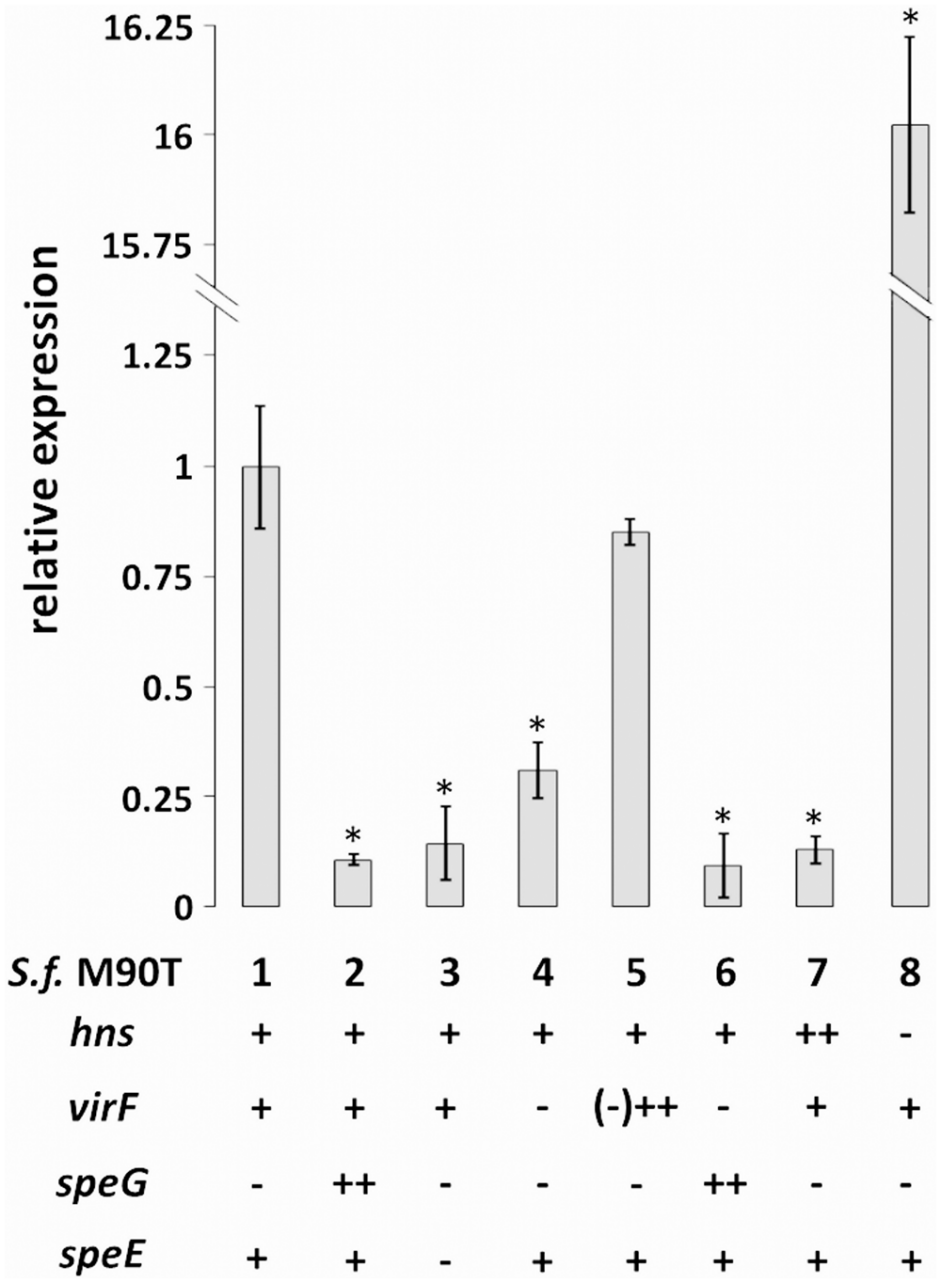
Relative *mdtJI* transcription in *Shigella* as monitored by *in vivo* Real-Time PCR. Quantitative analysis of *mdtJI* transcripts was performed by means of Real Time PCR assays using RNA extracted from the following *S*. *flexneri* strains: M90T wt (*hns* ^+^, *virF* ^+^, *speG* ^-^, *speE* ^+^; lane 1), M90T pULS37 (lane 2), M90T Ed (Δ*speE*; lane 3), M90T Fd (Δ*virF*; lane 4), M90T Fd pMYSH6504 (lane 5), M90T Fd pULS37 (lane 6), M90T pDIA510 (lane 7), M90T Hd (Δ*hns*, lane 8). All the strains were grown at 37°C in LB medium. The presence of a recombinant plasmid is always indicated by ++. At least three wells were run for each sample and the error bars display the calculated maximum (RQMax) and minimum (RQMin) expression levels that represent standard error of the mean expression level (RQ value); Student’s t tests were performed comparing the *mdtJI* relative expression in *S*. *flexneri* M90T wt strain with its derivatives, * denotes p < 0, 01.

A confirmation of the Northern analysis and a more accurate quantification of *mdtJI* transcription was obtained by Real Time PCR analysis (Fig 2). Indeed, as compared to the wt (Fig 2, lane 1), *mdtJI* transcription shows a more than 9-fold decrease in a *speG-*complemented strain (able to convert spermidine into acetylspermidine; Fig 2, lane 2). A similar effect, i.e. an about 7-fold decrease, is observed in a *S*. *flexneri* mutant that lacks the *speE* gene encoding the spermidine synthase (SpeE) (Fig 2, lane 3). Altogether, these data clearly indicate that in *Shigella* a decrease in intracellular spermidine strongly affects the expression of the *mdtJI* operon. This is in agreement with previous observations indicating that in *E*. *coli mdtJI* is activated in response to spermidine [20].

We investigated if the low expression of *mdtJI* in *E*. *coli* depends also on factors other than reduced spermidine content. Indeed, it is known that H-NS, one of the major nucleoid-associated proteins, is able to repress a large number of genes [42], including some encoding efflux pump subunits [43]. We therefore compared the expression of *mdtJI* in *E*. *coli* MG1655 and in its *hns*-defective derivative ULS504 (Table 1). Northern analysis shows that in the absence of H-NS (Fig 1, lane 1) *mdtJI* expression is increased by about 9-fold as compared to the wt (Fig 1, lane 2). Banking on this observation, we looked at the H-NS-dependency of *mdtJI* expression in *Shigella*. The results of the Northern assay (Fig 1, lanes 3 and 5), obtained by comparing *S*. *flexneri* M90T wt with a derivative transformed with a recombinant *hns* plasmid (M90T pDIA510), show that the overexpression of H-NS provokes a reduction of *mdtJI* mRNA, suggesting that H-NS negatively controls *mdtJI* expression also in *Shigella*. This is confirmed by Real Time PCR assays of *mdtJI* transcripts in *S*. *flexneri* M90T and in its *hns-*defective derivative M90T Hd: in the absence of H-NS *mdtJI* expression is increased more than 15-fold (Fig 2, lane 8).

The expression of the virulence genes in *Shigella* is greatly repressed by H-NS at low temperature [10,37]. At the host temperature VirF, the major regulator of the virulence gene cascade, is able to alleviate the H-NS-mediated repression of the *icsA* and *virB* promoters, thus allowing the full expression of the genes required for the invasion programme [44]. Recently transcriptomic analyses of an *E*. *coli* strain harbouring a multicopy *virF* plasmid have shown that VirF is able to induce expression of genes encoding housekeeping functions [16]. Thus VirF, besides being considered as an anti-H-NS protein [45], can be viewed also as a global regulator whose action is not limited to virulence systems. Therefore, we asked whether VirF is also able to relieve the H-NS-mediated repression of the *mdtJI* operon. By comparing *mdtJI* expression in *S*. *flexneri* M90T strains carrying or lacking the *virF* gene we find that *mdtJI* transcription is reduced about 3-fold in the absence of *virF* (Fig 1, lane 6; Fig 2, lane 4), and is restored to levels comparable to the wt in a *virF* strain containing a recombinant *virF* plasmid (M90T Fd pMYSH6504) (Fig 1, lane 9; Fig 2 lane 5). The crucial role played by spermidine and VirF in the activation of the *mdtJI* genes shows up clearly also by observing the severe repression of the *mdtJI* mRNA in a *virF*-depleted *S*. *flexneri* strain (M90T Fd) complemented with plasmids (pULS37, pULS13) carrying a functional *speG* gene (Fig 1, lanes 7 and 8; Fig 2, lane 6). Finally, taking account of the thermodependency of the expression of *virF* [10] in *Shigella*, we looked at the expression of *mdtJI* at different temperatures (30°C vs 37°C) in the same genetic background. The results of Real Time PCR assays, reported in S1 Fig, reveal that at 37°C the *mdtJI* mRNA is increased by about 1.8-fold as compared to 30°C, as expected on the higher expression of *virF* known to occur at 37°C [10,12].

Altogether, the initial Northern screening and the successive Real Time PCR quantification demonstrate that the *mdtJI* operon is silenced by H-NS and that its expression in a *Shigella* background relies on the higher level of spermidine and on the presence of VirF. It is known that polyamine-regulated proteins include a number of transcriptional factors [46]. In this context previous reports have shown that, at the early logarithmic growth phase in the absence of glutamate, polyamines slightly stimulate (1.5-fold) the synthesis of H-NS by enhancing the efficiency of translation while no effect has been observed on transcription [47]. As opposed to H-NS, at present the effect of polyamines on the expression of VirF is largely unknown. Thus, we examined the possible influence of spermidine on VirF expression both at the transcriptional and translational level. To this end, we constructed a *S*. *flexneri* M90T strain harboring a *virF* 3xFLAG tag allele on the pINV plasmid (Table 1) and we monitored, in strains carrying (pULS13, Table 1) or lacking a functional *speG* gene, the synthesis of the VirF protein and of the *virF* mRNA by Western blot analysis and, respectively, by Real Time PCR. The results are shown in S2 Fig and clearly indicate that, irrespective of the growth phase, the levels of the VirF protein and of the *virF* mRNA do not change when spermidine accumulates intracellularly.

### Binding of H-NS to the *mdtJI* promoter

**Fig 3:**
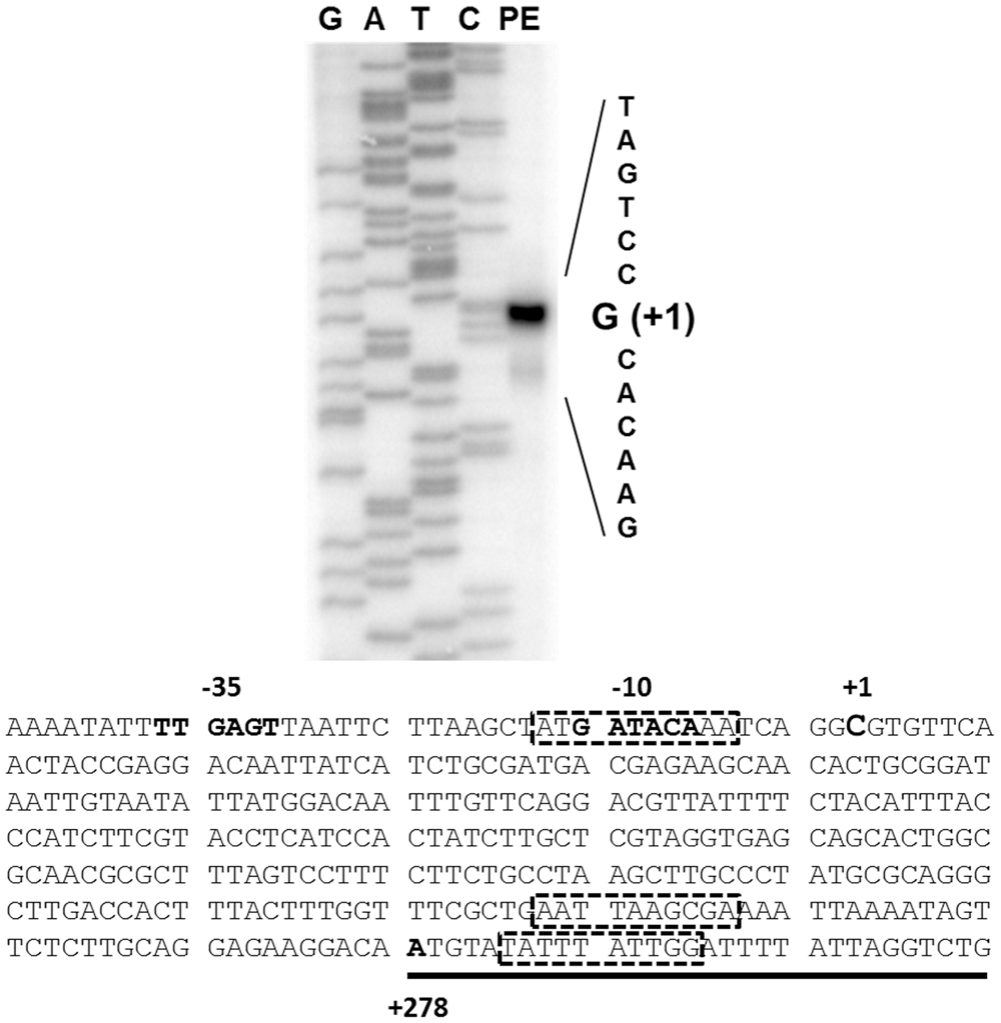
Identification of the *mdtJI* promoter region. The *mdtJI* transcription start site was located by primer extension of the *mdtJI* mRNA. The autoradiograph is the result of a typical experiment performed with the *mPE2* primer on RNA extracted from strain MG1655 pULS85 grown in LB medium at 37°C. Lanes G, A, T, and C show the sequencing ladder generated with the same primer. The sequence of the *mdtJI* regulatory region is reported in the lower panel. The bold sequence motifs represent the putative -10 and -35 hexamers of the *mdtJI* promoter and the transcription start site (+1). The MdtJ translation start codon is positioned at +278 (bold) and the coding sequence is underlined. The three putative H-NS binding sites are boxed with a dotted line.

We asked if the negative control exerted by H-NS on *mdtJI* is the result of a direct interaction with the promoter or if the repressive effect is mediated by other factors. We first identified the transcription start site of the *mdtJI* operon. A fragment extending about 500 bp upstream the MdtJ translation start site was cloned in a plasmid vector, obtaining pULS85 (Table 1). After RNA purification, a primer extension analysis of the transcripts generated under the control of the *mdtJI* promoter was carried out with the oligo *mPE1*, close to the *mdtJ* translation start. Considering that with this oligo the signal obtained originates from more than 300 nt upstream (data not shown), we repeated the assay by priming with oligo *mPE2*, located 200 nt upstream *mPE1*. As shown in Fig 3 this allowed to identify the transcription start site as a G positioned 278 bp upstream the MdtJ ATG codon and associated with -10 and -35 regulatory boxes. The significance of this long 5’-UTR is as yet unclear, though it is tempting to assume it may be relevant for the multifactorial regulation of the operon.

**Fig 4:**
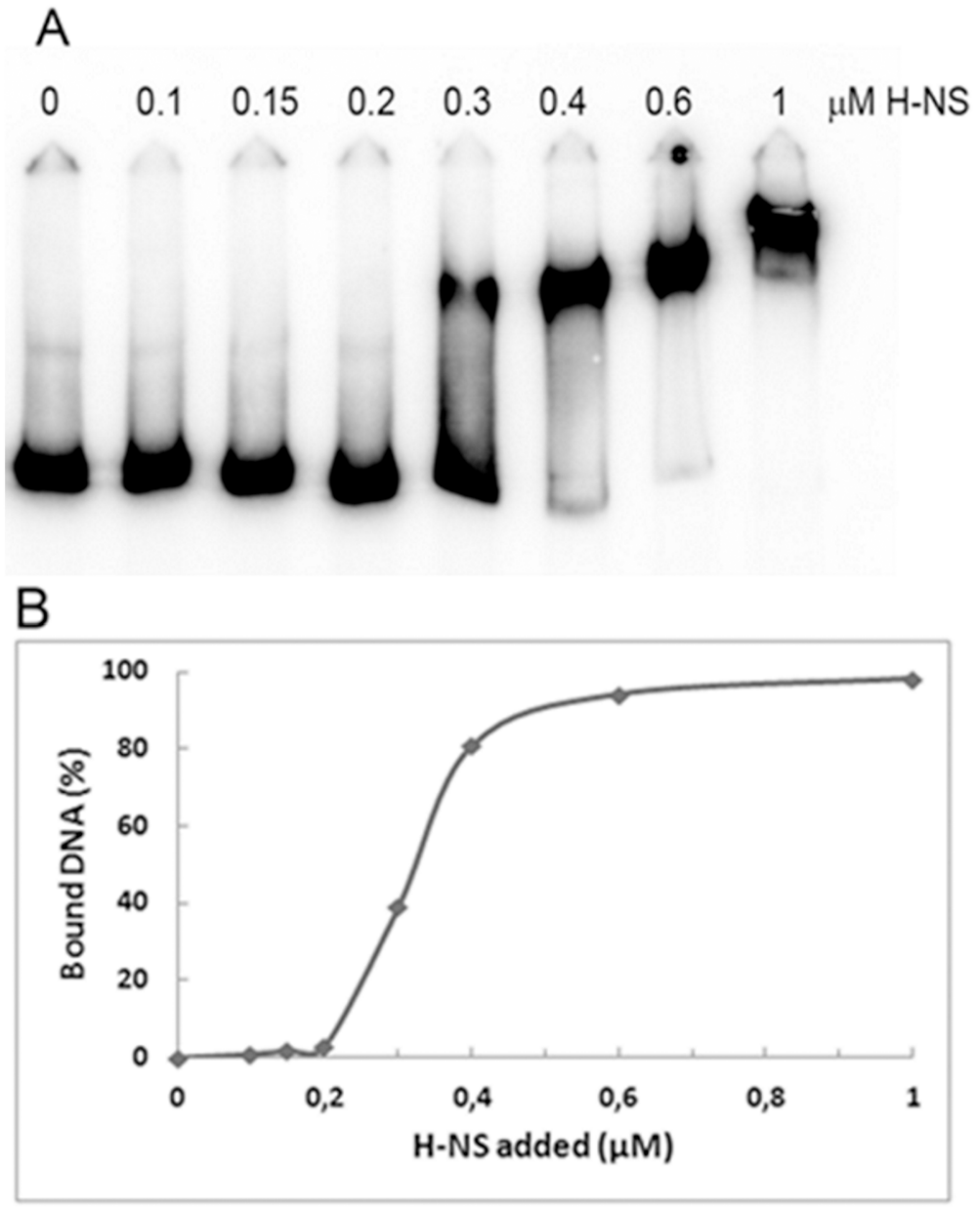
H-NS binds the *mdtJI* promoter region. (A) A ^32^P-labelled 588 bp DNA fragment covering the *mdtJI* promoter was incubated at 20°C for 10 min with the indicated concentrations of purified H-NS and subjected to an electrophoretic mobility shift assay. (B) After image quantization, the radioactivity associated with DNA-H-NS complexes, expressed as percentage of total DNA (bound plus free molecules), was plotted versus protein concentration.

Then, using the oligo pair *mdF/mdR* and pULS85 as template, we amplified a 588 bp DNA fragment containing the entire *mdtJI* promoter region and used it in Electrophoretic Mobility Shift Assays (EMSA). To this end, increasing amounts of purified H-NS were added to the DNA in the presence of a 10-fold excess of competitor poly(dI-dC). The result is shown in Fig 4. H-NS is clearly able to recognize the *mdtJI* promoter and to specifically interact with it, strongly suggesting that repression is due to direct binding. The retardation pattern is compatible with the progressive occupancy by H-NS of at least two binding sites, as denoted by the appearance of a lower mobility complex at the highest concentration of protein tested (Fig 4A, 1 μM H-NS). The quantitative analysis reveals that the H-NS binding affinity, expressed as protein concentration required to bind 50% of DNA, is slightly higher than at 0.3 μM (0.32 μM) H-NS, while almost complete retardation occurs in the range of 0.4–0.6 μM H-NS (Fig 4B). This result is in good agreement with the *in silico* analysis performed with the Virtual footprint/PRODORIC online tool [39] which indicates that H-NS has three potential binding sites within the *mdtJI* promoter. Interestingly, one of the predicted binding site overlaps the -10 consensus box (Fig 3) providing a possible explanation of *mdtJI* transcriptional silencing by H-NS.

**Fig 5:**
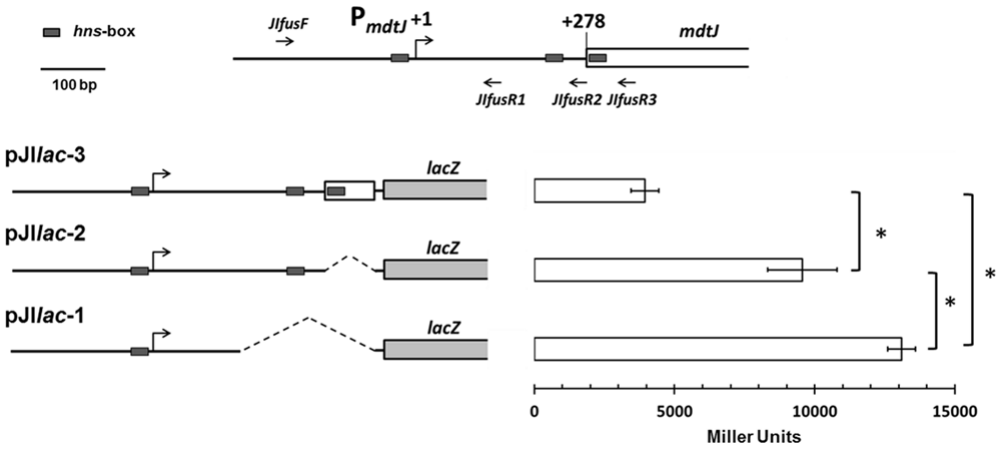
Analysis of the *mdtJI*::*lacZ* fusions. **Upper section**: Genetic organization of the *mdtJI* regulatory region. Arrows (*JIfusF*, *JIfusR1*, *JIfusR2*, and *JIfusR3*) indicate the primers used for amplifying fragments carrying different portion of the *mdtJI* regulatory regions. The small dark grey boxes represent the predicted H-NS binding boxes. Transcriptional (+1) and translational (+278) start sites are indicated. **Lower section**: The β-galactosidase activity of the *mdtJI*::*lacZ* fusions, carried by plasmids pJI*lac*-3, -2 and -1 was determined in *E*. *coli* ULS153. The values reported are expressed as Miller Units [31] and represent the average ± standard deviation of five independent experiments; * denotes p < 0, 01.

In an attempt to gain more information on the relevance of the predicted H-NS binding sites, we constructed three transcriptional *mdtJI*::*lacZ* gene fusions containing different portions of the *mtdJI* regulatory region. One construct, pJI*lac*-3, harbors a fragment carrying the three predicted H-NS sites. Constructs pJI*lac*-2 and pJI*lac*-1 carry fragments which lack one or, respectively, both promoter-distal H-NS boxes. The three constructs and the results of the β-gal assays are shown in Fig 5. We find that the “progressive” deletion of H-NS boxes “progressively” increases β-gal expression, as expected if H-NS is able to recognize its predicted binding sites in the region examined.

It is known that the DNA binding preference of H-NS includes DNA regions exhibiting tracts with significant intrinsic curvature [45,12]. Therefore, we analyzed the intrinsic DNA curvature of the 588 bp fragment containing the *mtdJI* promoter by means of an *in silico* approach. We used a software which we developed previously and which we have shown to produce accurate predictions [12,33]. The results, shown in S3 Fig, suggest that while the curvature profile shows, overall, some phasing with the map position of the putative H-NS boxes, the spreading of curvature values around the average curvature of the 588 bp *mdtJI* fragment is limited and cannot be assumed to indicate the presence of significantly bent DNA tracts.

### Functional characterization of MdtJI in *Shigella*

**Fig 6:**
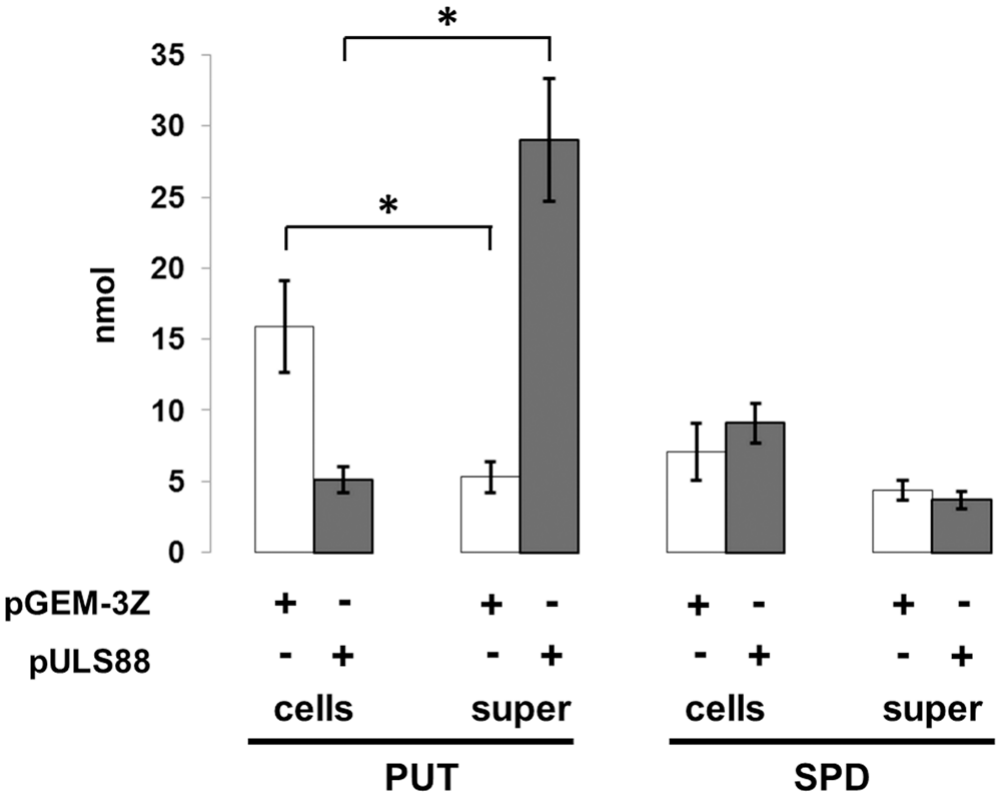
MdtJI facilitates putrescine secretion in *Shigella*. Analysis of the polyamine levels in *S*. *flexneri* M90T transformed with pULS88 carrying a functional *mdtJI* operon or with the pGEM-3Z backbone vector. Cells were grown in polyamine-free M9 complete medium to OD_600_ 0.7–0.8. Polyamines were extracted from cell lysates (cells) or from supernatants (super) as described in Material and Methods. Values reported are in nmoles/10^8^ cells (in the case of cellular extracts) or in ml of supernatant and represent the average ± standard deviation. PUT: putrescine; SPD: spermidine. Student’s t tests were performed comparing PUT and SPD concentrations from cell lysates or from supernatans of M90T pULS88 with the corresponding polyamine concentrations detected in M90T pGEM-3Z. * denotes p <0.01.

A number of efflux pumps have been identified in *E*. *coli*. They are often expressed at low levels, making their transport specificity difficult to assess, especially in the presence of a functional AcrAB complex [48], a major efflux system. A previous study [20], performed using *speG*-defective *E*. *coli* strains harboring the *mdtJI* operon on a multicopy vector, has shown that after 24 hours incubation in the presence of high concentrations of extracellular spermidine (2mM) the MdtJI complex catalyzes the excretion of spermidine. To understand to what extent the MdtJI pump is effectively involved in polyamine transport also in *Shigella*, bacterial cells were assayed for their capacity to excrete polyamines when grown in a polyamine-free medium. The results, obtained by means of HPLC assays (Fig 6), indicate that high-level expression of the *mdtJI* operon determines a 3-fold decrease of intracellular putrescine. The involvement of the MdtJI pump in putrescine secretion is confirmed by the observation that MdtJI promotes a 6-fold increase of putrescine in the supernatant. In contrast to putrescine, spermidine levels exhibit only a slight variation both within the cells and in the supernatants. Cadaverine and acetylspermidine were not detected since they are absent in *Shigella* due to the pathoadaptive silencing of the *cad* and *speG* genes [19]. All together, these data indicate that the MdtJI pump promotes the excretion of putrescine in *Shigella*, and imply that in the absence of the prolonged incubation under the high spermidine concentration adopted in other studies [20] MdtJI does not contribute to spermidine excretion. Taking account that putrescine is the spermidine precursor, lowering its intracellular level could contribute to limit spermidine accumulation and the consequent cytotoxic effect. The involvement of MdtJI in putrescine secretion has been observed also in the *E*. *coli* background [20]. In particular, the authors report that over-expression of *mdtJI* in *E*. *coli* Δ*speG* grown in a polyamine-free medium induces a 5-fold decrease of intracellular putrescine concentration.

**Fig 7:**
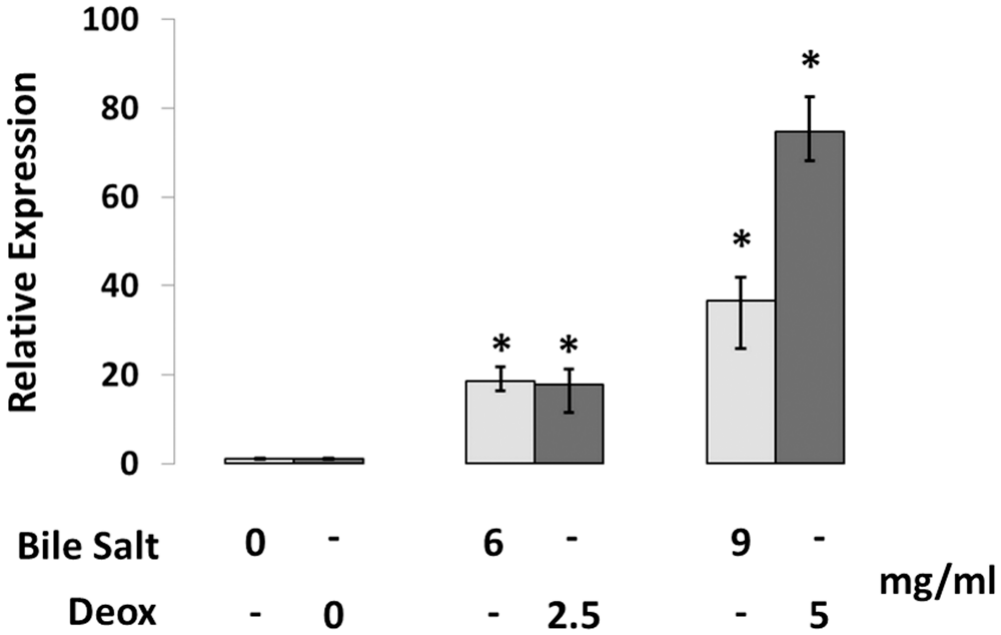
Bile stimulates *mdtJI* transcription. Real Time PCR assays showing transcription of *mdtJI* in the presence (6 or 9 mg/ml) or absence of bile salt (light grey bars), and transcription of *mdtJI* in the presence (2.5 or 5 mg/ml) or absence of sodium deoxycholate (Deox) (dark grey bars). At least three wells were run for each sample; error bars display the calculated maximum (RQMax) and minimum (RQMin) expression levels that represent standard error of the mean expression level (RQ value); Student’s t tests were performed comparing the *mdtJI* relative expression from treated strain with the relative expression from untreated strain, * denotes p < 0.01.

It has been previously reported [48] that in an *E*. *coli* K12 strain defective for the major efflux system (AcrAB), the MdtJI pump (YdgFE) confers a 4-fold increased resistance to deoxycholate (a major component of bile) when the pump is overexpressed and under the control of a strong promoter. Enteric bacteria are often resistant to the bactericidal effect of bile and the comparatively high concentration of bile salts in the intestine is often exploited by enteric pathogens to help identify their immediate environment, ensuring that the correct spatio-temporal requirements for the production of resistance and virulence factors are met [49]. Based on these observations, we analysed the sensitivity to deoxycholate and bile salts of *Shigella* strains expressing or lacking MdtJI. A spot test carried out on LB plates containing 2.5 or 5 mg/ml of deoxycholate, or 6 and 9 mg/ml bile salts, does not reveal significant growth differences (data not shown). Interestingly, when analyzing the expression of the *mdtJI* operon in response to bile salts or deoxycholate (Fig 7), we observe that both are able to significantly increase *mdtJI* transcription, suggesting that within the host bile contributes to stimulate the expression of the MdtJI pump.

Finally, we verified whether MdtJI confers increased antibiotic resistance to *Shigella*. Previous studies in *E*. *coli* [48] suggest that, a slight increase in the resistance to nalidixic acid and to phosphomycin is detectable when the *mdtJI* operon is cloned on a multicopy vector under the control of a strong promoter. The assays we have performed reveal that in *Shigella* the MdtJI complex, even if overexpressed, does not significantly affect the antibiotic sensitivity profile of the bacterium (data not shown).

## Discussion

*Shigella* is a highly adapted human pathogen causing bacillary dysentery, a life-threatening enteric disease [8]. It originated from *Escherichia coli*, its innocuous ancestor, through a variety of evolutionary steps, the major ones implying gain of functions facilitating the intracellular survival, and pathoadaptive mutations, i.e. loss of features hampering the full expression of the invasive phenotype [19,50]. While the acquisition of the large virulence plasmid (pINV) by *Shigella* has promoted, in a single step, the capacity to enter and multiply inside the highly specialized intracellular environment of the human intestinal mucosa [9,28,51], the loss of antivirulence functions has acted progressively to increase the pathogenic potential of these strains [19].

A paradigmatic case among pathoadaptive mutations is the inactivation of genes involved in the biosynthesis of polyamines. As compared to the commensal *E*. *coli*, cadaverine and acetylspermidine are not synthesized in *Shigella* [16,18]. The lack of cadaverine is known to increase the pathogenic potential of the bacterium in host tissues [18]. On the other hand, the lack of acetylspermidine, due to the absence of the gene (*speG*) encoding spermidine acetyltransferase (SAT), leads to a marked accumulation of spermidine, its precursor molecule [16]. High spermidine levels have been shown to promote the survival of *Shigella* under oxidative stress conditions such as those encountered during the infection of macrophages [16]. The overall relevance of polyamines in the life style of *Shigella* is exemplified by the fact that during host colonization the bacterium encounters environments with different polyamine concentrations. In the gastrointestinal tract, the rapid growth of intestinal epithelia implies a high demand for polyamines, and thus a comparatively high local accumulation of polyamines, as compared to other tissues in the body. In the present work, we have analyzed whether spermidine accumulation in *Shigella*, besides favouring the survival within the host, might also enhance the expression of the operon encoding the MdtJI efflux pump.

The transcriptional analysis we have performed indicates that in *Shigella* the expression of the *mdtJI* operon is about 3-fold higher than in *E*. *coli* (Fig 1). In particular, the results show that *mdtJI* expression is affected by the intracellular level of spermidine. Indeed, a reduction in spermidine content, obtained by introducing a functional *speG* gene or deleting the *speE* gene encoding spermidine synthase, results in a severe reduction of *mdtJI* expression. Further, our observations reveal that other factors besides spermidine affect *mdtJI* expression, notably H-NS, VirF and bile components, as discussed below.

We find (Figs 1 and 2) that, both in *Shigella* and in *E*. *coli*, a strong repressive effect is exerted by H-NS on the expression of the *mdtJI* operon. Several efflux pumps involved in multidrug resistance are derepressed in *E*. *coli hns* mutants, though in most cases this is an indirect effect due to the negative control exerted by H-NS on the genes encoding activators specific for drug exporters [43]. In particular, a crucial role is played by the H-NS controlled response regulator EvgA, which controls several MDR systems [52]. The gel-retardation assay shown in Fig 4 indicates that, as opposed to many other efflux pumps, the effect of H-NS on the *mdtJI* operon is due to a direct binding of the protein to the *mdtJI* regulatory region. The analysis of *mdtJI*::*lacZ* transcription fusions (Fig 5) suggests that the region downstream the transcription start site harbors sites relevant to the H-NS-mediated control. Indeed, deletions in this region strongly increase the activity of the reporter gene. An intriguing possibility is that H-NS might induce the formation of a repressor loop in this region, e.g. by bridging the promoter-proximal H-NS site with either one of the promoter-distal sites. The predicted overall DNA curvature profile of this region (S3 Fig) supports this possibility, though the deviation of individual curvature values from the average curvature of the region does not appear statistically significant. In terms of development prospects this issue will be addressed by footprinting assays, to accurately locate H-NS sites in this region, coupled with experimental mapping of bent DNA tracts.

Besides having a structural role in the condensation of the bacterial chromosome, H-NS is deeply involved in the selective silencing of a large number of genes, many of which have been acquired by horizontal gene transfer [42]. In the case of *Shigella*, it has been described that at low temperature H-NS represses all virulence genes located on the pINV plasmid, while at the host temperature the repressive effect is relieved by the VirF protein, which activates the promoters of the virulence gene cascade [10,14,15]. Recently, we have shown that VirF, besides acting as anti-H-NS protein [45], also activates genes whose products contribute to better withstand adverse conditions inside the host [16]. Here we show (Figs 1 and 2) that in *Shigella* the transcription of the *mdtJI* operon is reduced 2.4-fold in the absence of VirF. This indicates that VirF is likely able to contrast the repressive H-NS effect on the *mdtJI* promoter, as has been already described for the *virB* and *icsA* promoters [14,15]. In particular, in the latter cases, though the molecular mechanism adopted by VirF to alleviate H-NS is still unclear, it has been shown that a VirF binding site is located around the promoter consensus boxes and partially overlaps an H-NS site, thus suggesting the occurrence of a VirF/H-NS competition at this site.

The presence of VirF and the accumulation of spermidine justify the higher expression of the *mdtJI* operon in *Shigella* as compared to *E*. *coli*. This is confirmed by the observation (Figs 1 and 2) that complete silencing of *mdtJI* in *Shigella* can be obtained by the concomitance of two events, a decrease of the spermidine level reduction and the elimination of the VirF protein. The marked overexpression of *mdtJI* in a *Shigella hns* background (Fig 2) may be explained by the combined effect of the known increased expression of VirF in such a background [10,37] coupled with the lack of H-NS protein. The mechanism by which spermidine activates *mdtJI* transcription remains an open question. We have shown that the synthesis of VirF, which acts as *mdtJI* activator, is not influenced by intracellular spermidine accumulation (S2 Fig). This may indicate that other transcriptional factors, such as those known to belong to the polyamine modulon [46,47], might affect the regulation of *mdtJI*, creating an additional layer of regulatory complexity in this region.

The role played by VirF evidences that the acquisition of a new regulator by horizontal gene transfer is a crucial event for remodelling the transcriptional profile of the core genome, pushing the bacterium towards a more virulent phenotype. Moreover, our results confirm the current view [45] that pathogenic bacteria have evolved means, often involving re-deployment of existing regulatory proteins rather than employing dedicated antagonists, to counteract the silencing activity of H-NS and allow the expression of a given set of genes only under environmental conditions affecting the biological activity of those regulators.

Except for the housekeeping AcrAB-TolC pump, efflux pumps are generally expressed at low levels under ordinary laboratory growth conditions and in most cases their role is still undefined [48]. Initial evidence suggesting that the MdtJI pump acts as a potential drug transporter came from the observation that the *mdtJI* operon is able to confer resistance to deoxycholate (and, to a lesser extent, to nalidixic acid, fosfomycin and SDS) only in the presence of very specific conditions, i.e. when expressed on a high copy number plasmid under a strong promoter in an *E*. *coli* strain defective in AcrAB, the major drug efflux complex [48]. We observe that, though MdtJI does not contribute to increase *Shigella* survival in the presence of bile components (likely because of an intrinsic high resistance of *Shigella* to bile), the *mdtJI* genes are stimulated by bile salts and deoxycholate (Fig 7), suggesting that within the host MdtJI might contribute to bacterial adaptation to the host environment.

Previous studies in *speG*-defective *E*. *coli* mutants have shown that MdtJI is able, when expressed at high levels, to excrete spermidine after prolonged incubation in high spermidine containing medium [20]. Our results (Fig 6) indicate that in *Shigella* the *mdtJI* pump promotes the secretion of putrescine, the precursor of spermidine. In particular, the net increase (about 6-fold) of putrescine concentration in the supernatant observed following enhanced MdtJI expression is paralleled by a clear reduction (about 3-fold) of intracellular putrescine. Under the same conditions, the level of spermidine is not affected. These results are in agreement with previous observations [20] showing that in *E*. *coli* Δ*speG*, grown in a polyamine-free medium, the intracellular putrescine concentration decreases about 5-fold when *mdtJI* is overexpressed.

Our study represents a previously unavailable account of the overall complexity of the *mdtJI* regulatory region, highlighting the major factors which underlie the silencing of the *mdtJI* pump in *E*. *coli* and its expression in *Shigella*. While deeper molecular investigations are required to fully solve the structure and function of the *mdtJI* regulon at the molecular level, altogether our observations, besides giving new information on the regulation of *mdtJI* in *Shigella*, allow to envision a novel functional role of the MdtJI complex. It is well known that excessive spermidine accumulation has toxic effects on bacterial cells [53]. *Shigella* typically lacks *speG*, and hence the capacity to transform spermidine into its inert form, acetylspermidine. Spermidine levels within *Shigella* are therefore increased and it is reasonable to assume that to avoid an excess of intracellular spermidine the bacterium has developed effective countermeasures. In this context, considering that putrescine is the precursor of spermidine, the secretion of putrescine by the spermidine-induced MdtJI complex may be crucial to prevent toxicity by spermidine over-accumulation. This would confer on the MdtJI complex the significance of a safety valve allowing *Shigella* to accumulate spermidine to a level optimally suited to survival within infected macrophages without affecting bacterial cell viability.

## Supporting Information

S1 Fig *mdtJI* expression at different temperatures. Quantitative analysis of *mdtJI* transcripts was performed by means of Real Time PCR assays using RNA extracted from *S*. *flexneri* strain M90T (Table 1) grown at 30°C and at 37°C in LB medium. At least three wells were run for each sample and the error bars display the calculated maximum (RQMax) and minimum (RQMin) expression levels that represent standard error of the mean expression level (RQ value); Student’s t tests were performed comparing the *mdtJI* relative expression in *S*. *flexneri* M90T strain grown at 30°C with that in the same strain grown at 37°C, * denotes p<0,01.
(TIF)

S2 Fig *VirF* expression in response to spermidine. The effect of spermidine on the transcription and translation of *virF* was performed using the *speG*-defective *S*. *flexneri* M90T *virF*-FT (carrying a *virF* 3xFLAG tag fusion) or its derivative complemented with a *speG* recombinant plasmid (pULS13). Cells were grown in LB medium at 37°C to OD_600_ 0.2 or 0.6. Upper section: quantitative analysis of *virF* mRNA performed by Real Time PCR. At least three wells were run for each sample. The error bars display the calculated maximum (RQMax) and minimum (RQMin) expression levels that represent standard error of the mean expression level (RQ value). Lower section: immunodetection of VirF-FT. Western blots were probed with anti FLAG antibodies and successively treated with a secondary horseradish peroxidase-conjugated antibody. The relative quantification of proteins was performed as described in Materials and Methods.
(TIF)

S3 Fig Computer-generated prediction of intrinsic DNA curvature of a 588 bp fragment encompassing the *mdtJI* promoter region. In essence, the software used slides a scanning window in 1 bp increments along the DNA sequence and computes the curvature of the DNA axis. The values are then normalized as end-to-end distance (Å)/double helix turn (assumed to inversely correlate with the intrinsic bending of the double helix axis) and plotted against a map of the fragment. The dotted line corresponds to the average curvature of the fragment minus 1.96 x SD (std.deviation), i.e. values lower than this threshold are assumed to indicate tracts endowed with significant intrinsic DNA bending as compared to the rest of the fragment. Black boxes: -35 and -10 consensus elements. White boxes: putative H-NS binding sites. +1: transcription start. +278: translation start.
(TIF)

S1 Table Oligos used in this study. (DOCX)
