# Supplementary figures and images for: Multifactor Regulation of the MdtJI Polyamine Transporter in Shigella
Source: PLoS One. 2015 Aug 27;10(8):e0136744. doi: 10.1371/journal.pone.0136744 (PMC4636849; doi:10.1371/journal.pone.0136744)

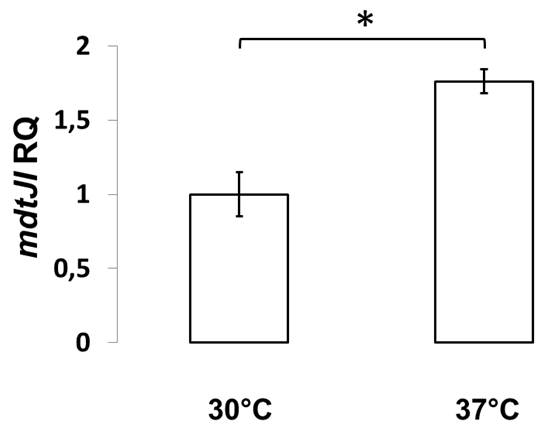

Supplement: S1 Fig — Quantitative analysis of mdtJI transcripts was performed by means of Real Time PCR assays using RNA extracted from S. flexneri strain M90T (Table 1) grown at 30°C and at 37°C in LB medium. At least three wells were run for each sample and the error bars display the calculated maximum (RQMax) and minimum (RQMin) expression levels that represent standard error of the mean expression level (RQ value); Student’s t tests were performed comparing the mdtJI relative expression in S. flexneri M90T strain grown at 30°C with that in the same strain grown at 37°C, * denotes p<0,01. (TIF) [file pone.0136744.s001.tif]

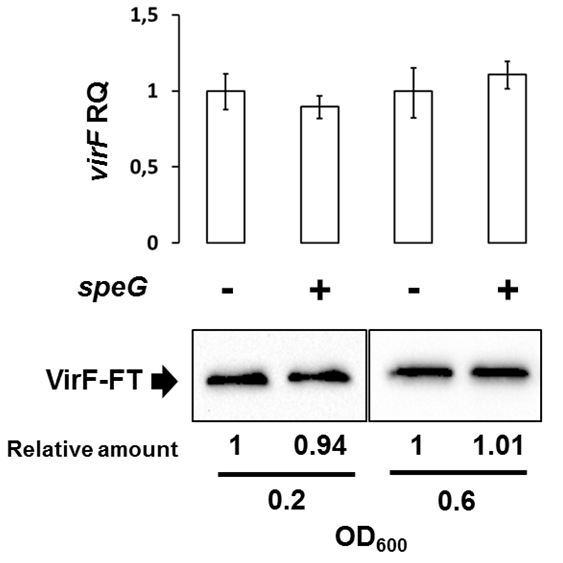

Supplement: S2 Fig — The effect of spermidine on the transcription and translation of virF was performed using the speG-defective S. flexneri M90T virF-FT (carrying a virF 3xFLAG tag fusion) or its derivative complemented with a speG recombinant plasmid (pULS13). Cells were grown in LB medium at 37°C to OD600 0.2 or 0.6. Upper section: quantitative analysis of virF mRNA performed by Real Time PCR. At least three wells were run for each sample. The error bars display the calculated maximum (RQMax) and minimum (RQMin) expression levels that represent standard error of the mean expression level (RQ value). Lower section: immunodetection of VirF-FT. Western blots were probed with anti FLAG antibodies and successively treated with a secondary horseradish peroxidase-conjugated antibody. The relative quantification of proteins was performed as described in Materials and Methods. (TIF) [file pone.0136744.s002.tif]

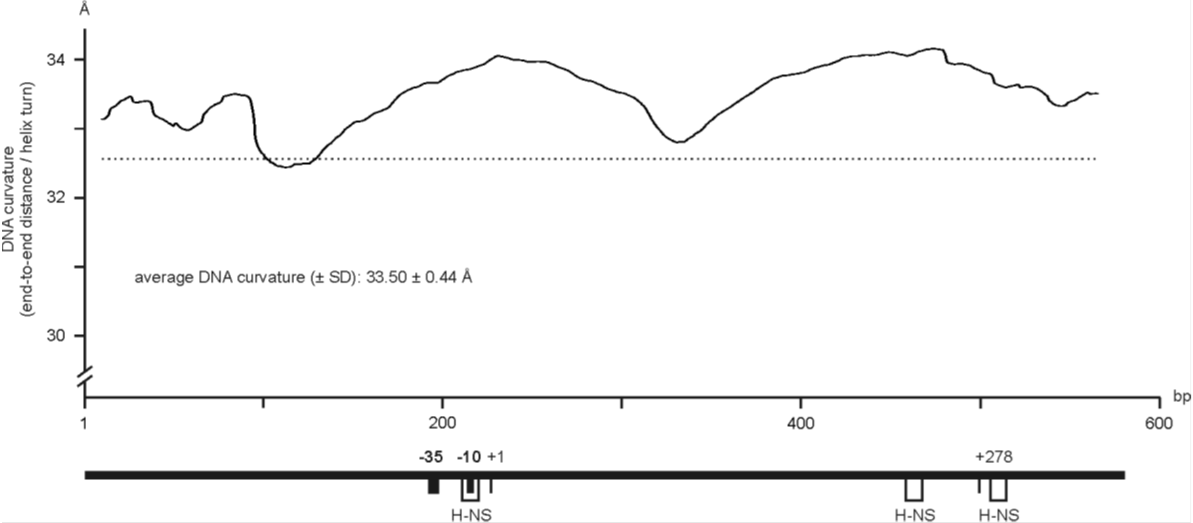

Supplement: S3 Fig — In essence, the software used slides a scanning window in 1 bp increments along the DNA sequence and computes the curvature of the DNA axis. The values are then normalized as end-to-end distance (Å)/double helix turn (assumed to inversely correlate with the intrinsic bending of the double helix axis) and plotted against a map of the fragment. The dotted line corresponds to the average curvature of the fragment minus 1.96 x SD (std.deviation), i.e. values lower than this threshold are assumed to indicate tracts endowed with significant intrinsic DNA bending as compared to the rest of the fragment. Black boxes: -35 and -10 consensus elements. White boxes: putative H-NS binding sites. +1: transcription start. +278: translation start. (TIF) [file pone.0136744.s003.tif]
