# Supplementary material for: Multifactor Regulation of the MdtJI Polyamine Transporter in Shigella
Source: PLoS One. 2015 Aug 27;10(8):e0136744. doi: 10.1371/journal.pone.0136744 (PMC4636849; doi:10.1371/journal.pone.0136744)
Supplement: S1 Table — (DOCX) [file pone.0136744.s004.docx]

| **Primer** | **Sequence 5’ – 3’** |
| --- | --- |
| *JIdF* | GGGAAATGGCGGCTTTATTTTAATGCTGGTGATGATTTGTGTAGGCTGGAGCTGCTTCG |
| *JIdR* | TTAACCGTTGACCAAACAAGATCCAACCTGCGGCTAATTCCGGGGATCCGTCGACC |
| *JIfusF* | NNNGGATCCATCTCGGCGGCAAAACGGA |
| *JIusR1* | NNNGGATCCCGAGCAAGATAGTGGATGAGG |
| *JIfusR2* | NNNGGATCCATTGTCCTTCTCCTGCAAGAG |
| *JIfusR3* | NNNGGATCCCACTGACGCTCGCCCATTTC |
| *ftFF* | TAACTCCAAAGAAATTTTACTTATATCATAAAAAATTTGACTACAAAGACCATGACGG |
| *ftFR* | TCAATCACAGCTTTCACGACCTTAATACGCAACTCTCATATGAATATCCTCCTTAG |
| *dff* | ATGCAAAAAGGTGTTCAATGACGGTTAGCTCAGGCAATGAGTGTAGGCTGGAGCTGCTTC |
| *dfr* | GACGCCATCTCTTCTCGATGTTTTTTTCAACAATCTTCCATTCCGGGGATCCGTCGACC |
| *mdF* | NNNGGATCCCTCGGCGGCAAAACGGATA |
| *mdR* | NNNGGATCCGACGCTCGCCCATTTCATTG |
| *JIF* | NNGACGTCTCAGGCGTGTTCAACTACCGA |
| *JIR* | NNNGAATTCGCGACAAGCTGGATCAACTG |
| *mtpF* | tatgcgctgtgggaaggtatc |
| *mtpR* | aataccaaacccgccccacaa |
| *mJIf* | TTCGCCGTTAAAAAATGC |
| *mJIr* | ACCTTCCCACAGCGCATAAG |
| *nusAF* | CGTAGGCACATTCGAACAACG |
| *nusAR* | CCTGGTACAGCAAGTTGCG |
| *virFQL* | GCCTTTCGGCAAAAGAAAGAT |
| *virFQR* | TCGAAGTATATAAAGCTTCCTCATCAGA |
| *mPE1* | gacgctcgcccatttcattg |
| *mPE2* | GTCGATGAGGTACGAAGATG |
